# Benefits of robust optimization in comparison with PTV‐based planning approach to overcome beam positioning uncertainties in spot‐scanning proton therapy

**DOI:** 10.1002/acm2.70355

**Published:** 2025-11-14

**Authors:** Vasilii A. Kiselev, Aleksander P. Chernyaev, Mikhail A. Belikhin, Rail R. Zamaltdinov, Sergei A. Vladimirkin, Sergei E. Gritsenko, Aleksey V. Nechesnyuk

**Affiliations:** ^1^ FSBI "Federal Scientific Clinical Center for Medical Radiology and Oncology" of FMBA of Russia Dimitrovgrad Russian Federation; ^2^ Lomonosov Moscow State University Moscow Russian Federation; ^3^ Dmitry Rogachev National Medical Research Center of Pediatric Hematology Oncology and Immunology Moscow Russian Federation

**Keywords:** proton therapy, quality assurance, robust optimization, treatment planning, uncertainties

## Abstract

**Background and purpose:**

The inherent uncertainties associated with proton therapy (PT) may present a challenge to the optimal clinical utilization of this radiotherapy modality. The possibility to overcome the uncertainties associated with temporal fluctuations in some parameters of the PT system by selecting an additional clinical target volume to planning parget volume (CTV‐PTV) margin or by modifying the planning approach was suggested. The objective of this work was to verify the sufficiency of the calculated additional margin for overcoming beam positioning uncertainties in spot‐scanning PT.

**Methods:**

Treatment plans for eight patients were calculated using two methods (robust optimization and PTV‐based approaches) and evaluated with regard to dose‐volume histogram (DVH)‐based dosimetric metrics. The robustness of each plan was evaluated under six scenarios in linear directions ±4 mm and two scenarios with a ±3.5% range uncertainty. A comparison between robust optimization and PTV‐based approaches was performed in the context of additional 1‐mm setup uncertainty associated with facility‐specific beam uncertainties.

**Results:**

No statistically significant difference was identified between the initial plans of the PTV‐based and robust approaches with regard to investigated dosimetric metrics. But robust optimization shows better results in comparison to PTV approach in majority of investigated scenarios. CTV was fully encompassed by the 100% dose in the plans created using the robust optimization approach. The Robust approach demonstrated superior dose conformity to the CTV, both for the 100% and 95% prescribed doses. Although both the PTV and Robust approaches permitted homogeneity in all scenarios, the superiority of the Robust approach was evident.

**Conclusions:**

It is necessary to consider the facility‐specific beam uncertainty as additional margin during treatment planning in PT. It was demonstrated that the robust optimization approach is an effective method for eliminating beam‐related facility‐specific uncertainties order of 1 mm in comparison to the PTV planning approach.

## 1 INTRODUCTION

Proton therapy (PT) is becoming an increasingly prevalent method of radiotherapy. Despite the numerous advantages of protons, several uncertainties remain, the omission of which may result in unfavorable outcomes for the patient. Anatomical changes along the proton path, whether occurring during a single fraction or throughout the entire course of PT, have the potential to disrupt the conformity of the dose distribution. This is particularly relevant for localizations of the irradiated volumes, such as the lung,^1^ head, and neck,^2^ which are characterized by complex inhomogeneity. These factors increase the probability of delivering high doses to normal tissue distal to the target.

It is anticipated that uncertainties associated with patient setup errors will be offset by the implementation of robust optimization. However, this concept doesn't take into account facility‐specific beam‐related uncertainties during beam delivery. Daily beam measurements of the pencil beam scanning (PBS) system have revealed fluctuations in spot positions, spot size, energy selection system performance, and coincidence of proton and x‐ray systems.^3^, ^4^ Insufficient consideration of these fluctuations may result in an inadequate selection of CTV‐PTV margins, either in PTV planning or using robust optimization.

Range errors are typically associated with alterations in the patient's anatomy and inaccuracies in the definition of CT numbers.^5^, ^6^, ^7^ Additionally, numerous studies have examined the impact of systematic and random errors in spot position delivery on dose distribution.^8^, ^9^ A number of studies have demonstrated that the implementation of beam‐specific PTV^10^ or robust optimization^11^, ^12^ can yield superior efficiency when compared to the conventional homogenous PTV approach.

The margin accounting for beam positioning fluctuations of the specific PT system has been previously calculated^13^ in our institution and assumed to be approximately 1 mm. This calculation was derived subsequent to an investigation into PBS system beam positioning parameters' deviations that spanned a 1‐year period. The investigation entailed an examination of errors pertaining to spot position, proton versus x‐ray central axis coincidence, and range selection system. The margin accounting for beam positioning fluctuations in three dimensions was evaluated as well.

In order to investigate the ability of PTV‐based and robust optimization approaches to resist beam‐related errors, an additional 1‐mm offset was introduced in the robustness analysis, in order to simulate the facility‐specific error in beam range and spot position.

## 2 METHODS

In this study, the PT system Proteus Plus (IBA, Louvain‐la‐Neuve, Belgium) was employed. This system utilizes a C235‐V3 cyclotron and four treatment rooms, two of which are equipped with PBS gantries. All beam parameter measurements and plan calculations were conducted for gantry treatment rooms utilizing PBS. The energy range available for treatment was 100–226.1 MeV. Gantries were able to rotate a full 360 degrees (±180 degrees).

A total of eight patients with diverse brain tumors who underwent PT at the Proton Center of FSBI «Federal Scientific Clinical Center for Medical Radiology and Oncology» of FMBA of Russia (Dimitrovgrad, Russian Federation) between 2022 and 2024 were selected for this study. Patient selection criteria were CTV volume of 20–80 cc, CTV location a minimum of 20 mm from body surface, 20 mm from the organs at risk (OAR), 20 mm from air cavities. The CTV volumes of patients included in this study varied from 23.0 to 79.7 cc (with a median value of 43.8 cc). During the actual treatment planning stage, the PTV margin value was determined to be isotropic at 3 mm in all cases. All patients underwent computed tomography (CT) scans with a slice thickness of 1 mm using the Brilliance BigBore (Philips, Amsterdam, Netherlands) for the delineation of the CTV and OARs. The delineation was performed with Monaco 5.11 (Elekta Limited, Stockholm, Sweden).

This study was conducted in accordance with the ethical standards set forth in the Declaration of Helsinki and received approval from the institutional review board (IRB) at the authors’ clinical institution. The requirement for written informed consent was waived by the IRB on the grounds of the retrospective nature of the study.

The intensity modulated proton therapy (IMPT) plan calculation was performed using Pinnacle 16.2^14^ (Philips, Amsterdam, the Netherlands) treatment planning system (TPS). Two approaches were introduced for plan optimization: PTV‐based and Robust. In the PTV‐based approach, a margin accounting for 3‐mm setup uncertainty and 3.5% range uncertainty was applied to the CTV.^10^ In contrast, the Robust approach utilized robust optimization on the CTV, incorporating a 3‐mm setup uncertainty and a 3.5% range uncertainty. Consequently, it was assumed that the setup and range uncertainty margin was taken into account in both approaches. It was assumed that relative biological effectiveness (RBE) was constant and equal to 1.1. The calculation grid size was 1×1×1 mm^3^. All treatment plans comprised two fields with a minimum of 30 degrees between them. The prescribed dose was 60 Gy (RBE) in 30 fractions. After optimization, each plan was normalized to ensure that a CTV coverage was V100 = 100%.

A total of 18 dose distribution scenarios were generated for each patient: two initial plans with PTV‐based and the Robust approaches and 16 robustness evaluation scenarios (eight scenarios for each approach). The robustness of each initial plan was evaluated under following scenarios: six scenarios with a 4‐mm setup uncertainty in lateral, longitudal, and vertical directions and two with a 3.5% range uncertainty resulting in a total of 144 scenarios for analysis (72 scenarios for each planning approach). A 4‐mm setup uncertainty in robustness evaluation scenarios was chosen by summation of 3‐mm patient setup uncertainty used in the clinic and 1‐mm beam uncertainty. The objective was to assess the ability of these approaches to overcome potential beam positioning uncertainties assumed to be 1 mm.

After the robustness evaluation, each scenario was analyzed for the following DVH‐based metrics:

1. V100%[%]—the CTV covered by 100% of the prescribed dose;
2. V95%[%]—the CTV covered by 95% of the prescribed dose;
3. V100%[cc]—the volume (cc) covered by 100% of the prescribed dose;
4. V95%[cc]—the volume (cc) covered by 95% of the prescribed dose;
5. D2%[Gy_RBE]—the dose (RBE) that covers 2% of the CTV;
6. D50%[Gy_RBE]—the dose (RBE) that covers 50% of the CTV;
7. D98%[Gy_RBE]—the dose (RBE) that covers 98% of the CTV.

A homogeneity index (HI) and conformity indices (CI_100_ and CI_95_) were calculated for each scenario. The HI was calculated according to the ICRU 83 recommendations.^15^ Better homogeneity was achieved when the HI was closer to 0. CI_100_ and CI_95_ were defined according to the approach proposed by Paddick.^16^ Better conformity was achieved when the CI was closer to 1.

Mann–Whitney *U* test was used to compare the initial plans obtained by the PTV‐based and Robust approaches by the CTV coverage, HI, CI_100_, and CI_95_ values to determine if these plans were clinically equal. Mentioned values were also compared by Mann–Whitney *U* test in all robustness analysis scenarios to compare their clinical quality.

## 3 RESULTS

**FIGURE 1:**
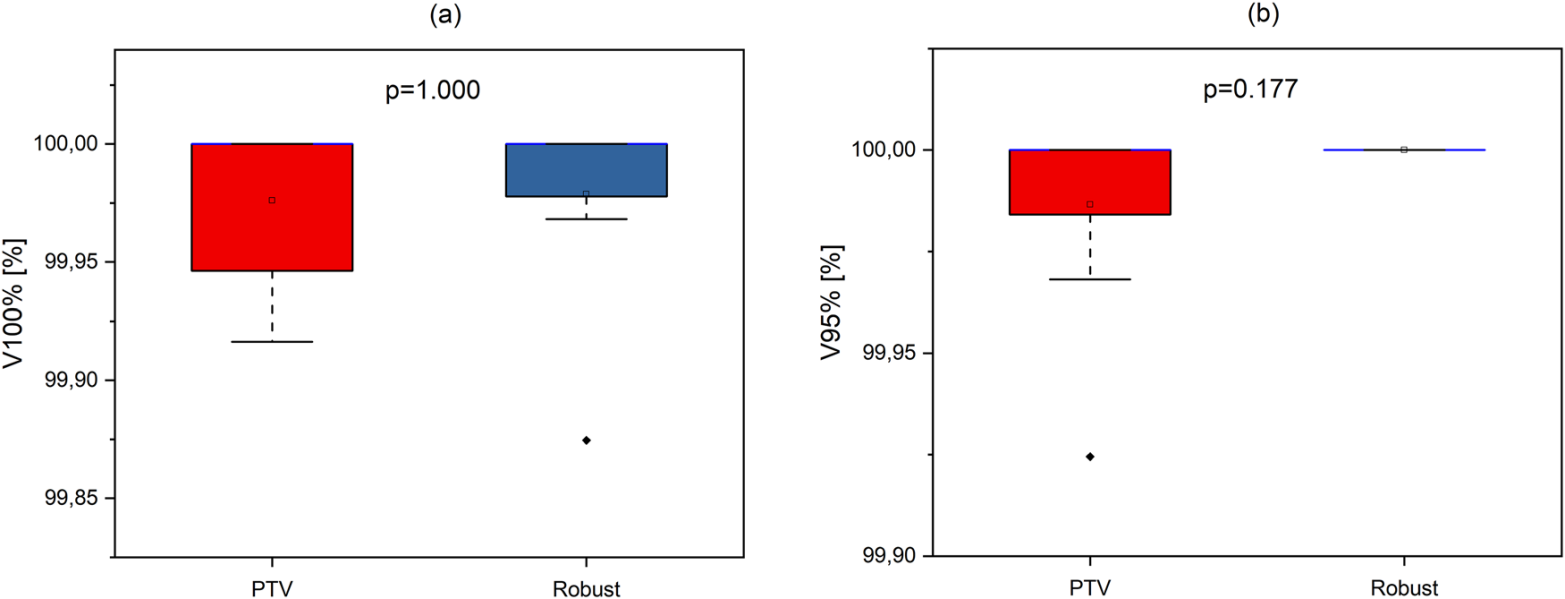
Boxplots illustrating percentage coverage of CTV by (a) 100% (V100%[%]) and (b) 95% (V95%[%]) of prescribed dose in initial plans for different approaches. The open square shows mean value. The blue center line denotes the median value (50th percentile), while the boxes contain the 25th–75th percentiles of dataset. The black whiskers mark the 1.5 interquartile range, and values beyond these bounds are considered outliers, marked with rhombus black dots.

**FIGURE 2:**
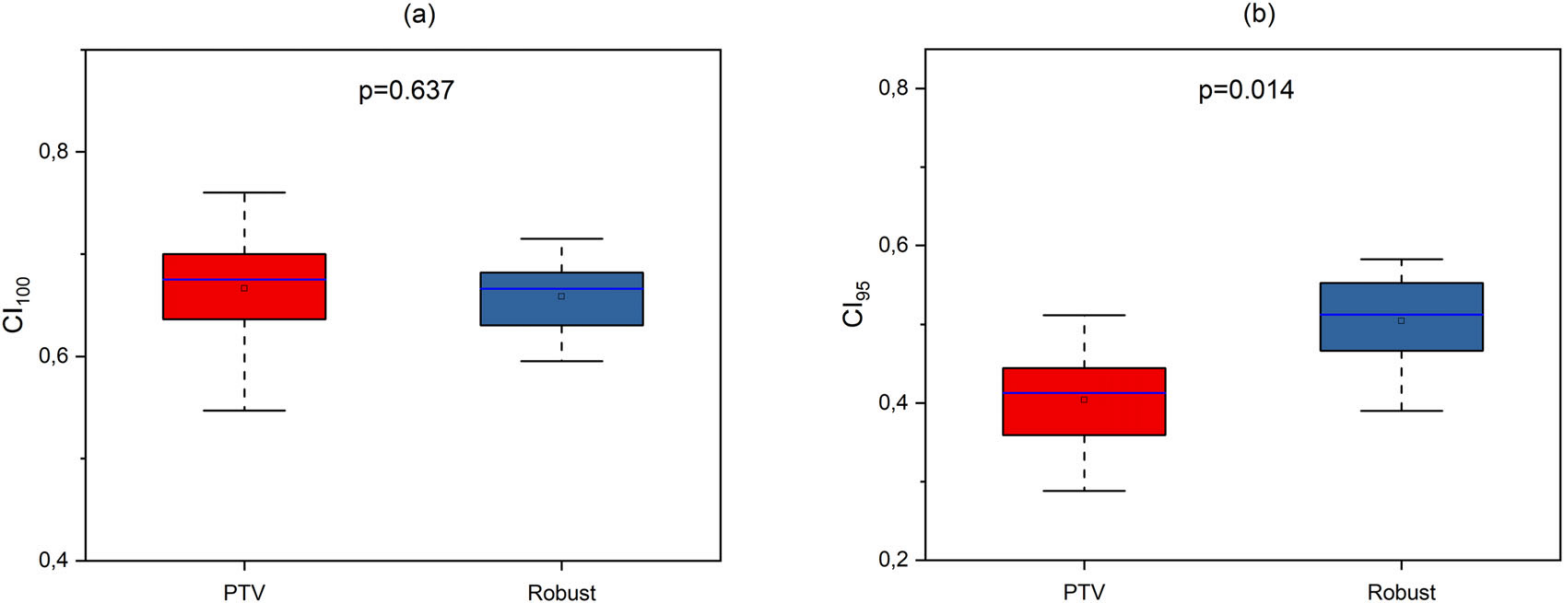
Boxplots illustrating CI calculated for (a) 100% isodose (CI_100_) and (b) 95% isodose (CI_95_) in initial plans for different approaches. The open square shows mean value. The blue center line denotes the median value (50th percentile), while the boxes contain the 25th–75th percentiles of dataset. The black whiskers mark the 1.5 interquartile range.

**FIGURE 3:**
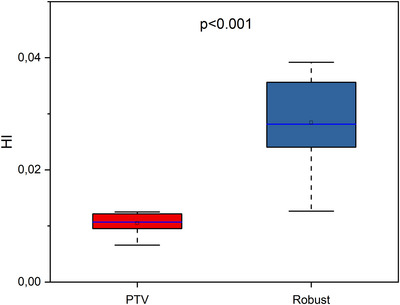
Boxplots illustrating HI in initial plans for different approaches. The open square shows mean value. The blue center line denotes the median value (50th percentile), while the boxes contain the 25th–75th percentiles of dataset. The black whiskers mark the 1.5 interquartile range.

An evaluation of V100%[%], V95%[%], CI_100_, CI_95_, and HI values of initial plans in different planning approaches is presented in Figures 1, 2, and 3.

As shown in Figure 1, no statistically significant difference was observed between the initial plans for the PTV‐based and Robust approaches with regard to V100%[%] (*p* = 1.000) and V95%[%] (*p* = 0.177). Figure 2 illustrates no statistically significant difference in CI_100_ (*p* = 0.637), but there is statistically significant difference in CI_95_ (*p* = 0.014). Figure 3 shows statistically significant difference in distribution homogeneity within the CTV (*p* < 0.001). In addition, the initial plans of the PTV‐based approach showed the superior CI_95_ and HI values. Median values of CI_95_ were 0.41 for PTV plans and 0.51 for Robust plans. Median values of HI were 0.01 for PTV plans and 0.03 for Robust plans.

**FIGURE 4:**
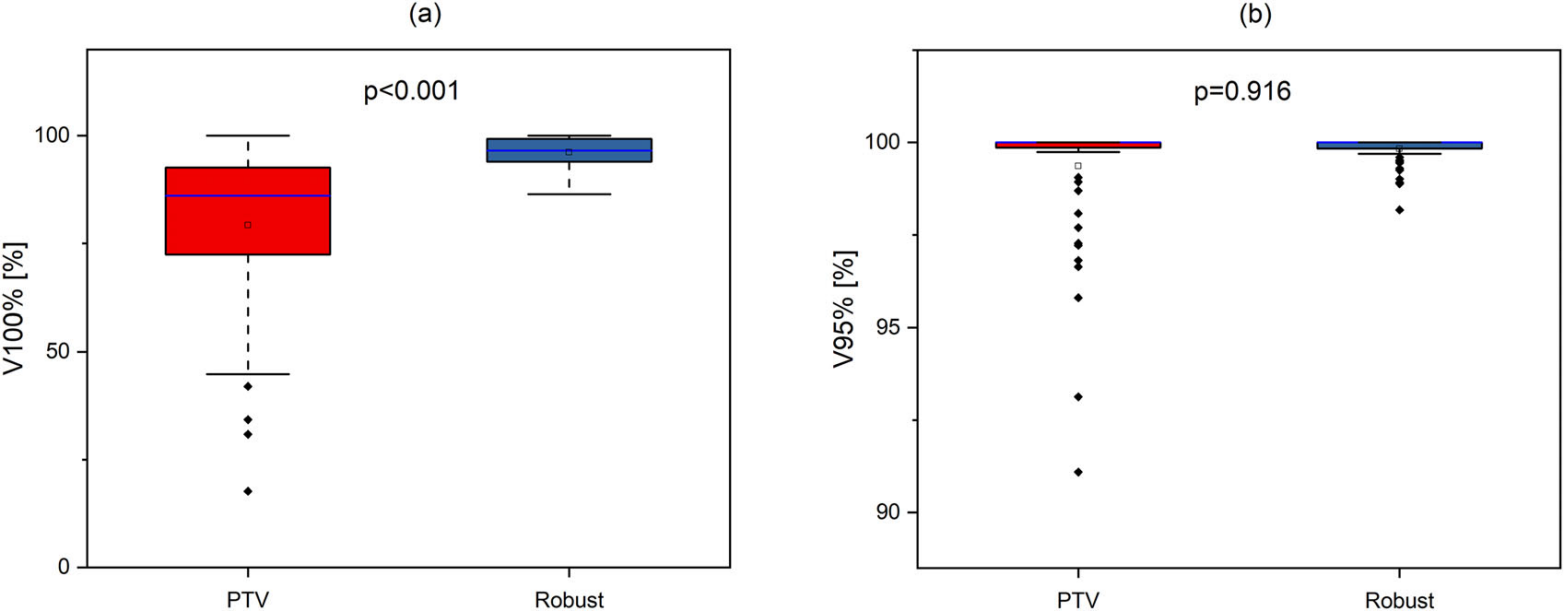
Boxplots illustrating percentage coverage of CTV by (a) 100% (V100%[%]) and (b) 95% (V95%[%]) of prescribed dose in robustly evaluated plans for different approaches. The open square shows mean value. The blue center line denotes the median value (50th percentile), while the boxes contain the 25th–75th percentiles of dataset. The black whiskers mark the 1.5 interquartile range, and values beyond these bounds are considered outliers, marked with rhombus black dots.

**FIGURE 5:**
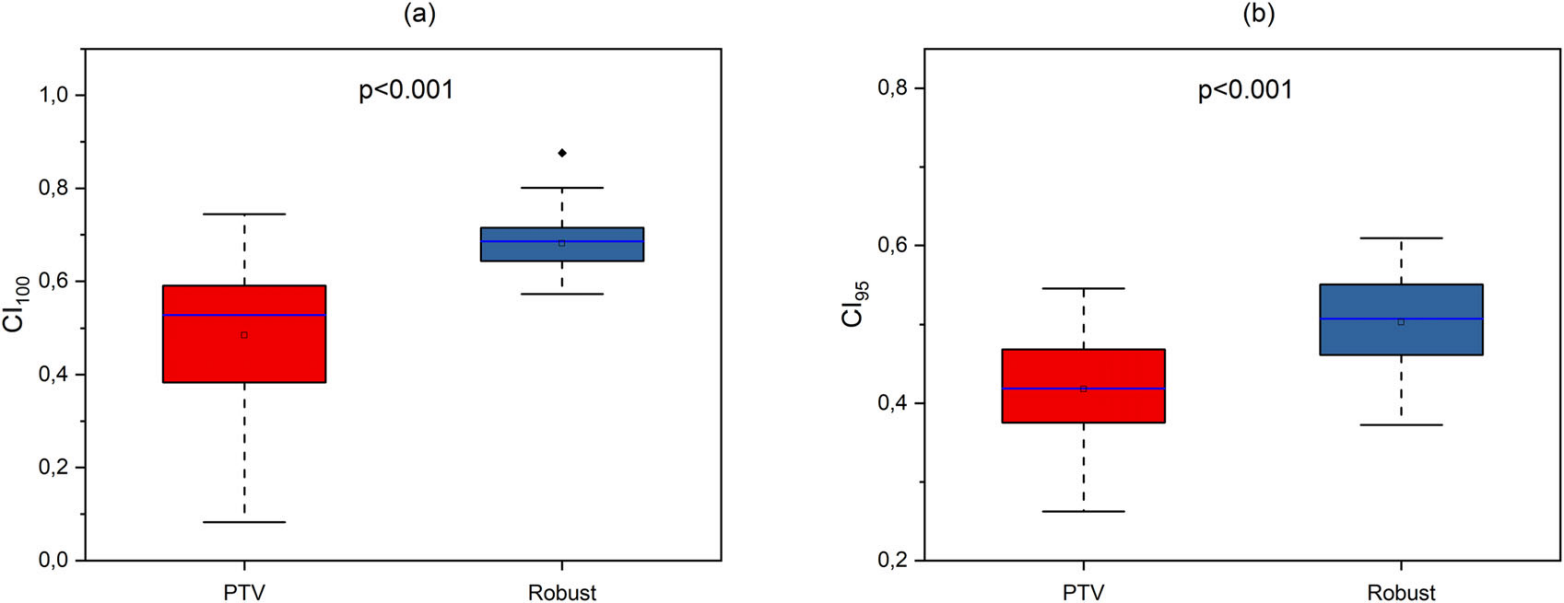
Boxplots illustrating CI calculated for (a) 100% isodose and (b) 95% isodose in robustly evaluated plans for different approaches. The open square shows mean value. The blue center line denotes the median value (50th percentile), while the boxes contain the 25th–75th percentiles of dataset. The black whiskers mark the 1.5 interquartile range. CI,

**FIGURE 6:**
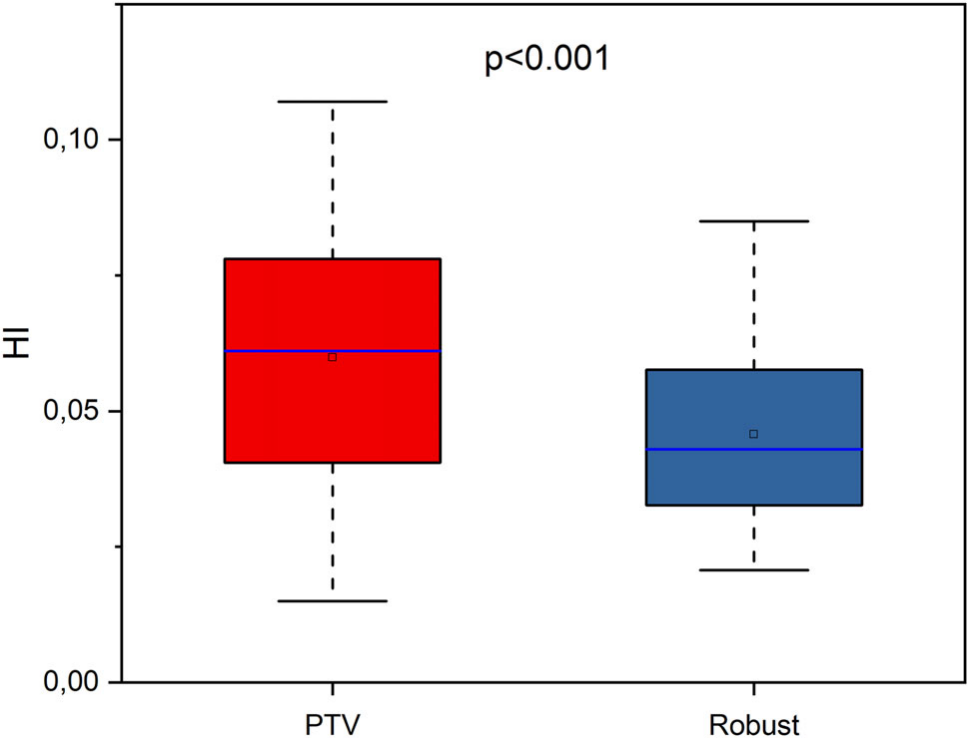
Boxplots illustrating HI in robustly evaluated plans for different approaches. The open square shows mean value. The blue center line denotes the median value (50th percentile), while the boxes contain the 25th–75th percentiles of dataset. The black whiskers mark the 1.5 interquartile range. HI,

An evaluation of CTV coverage, HI, CI_100_, and CI_95_ values of robustly evaluated plans in different planning approaches is presented in Figures 4, 5, and 6. The V100%[%] coverage was higher in 57 out of 64 (89.1%) scenarios when the Robust approach was applied. The V95%[%] coverage was better in 23 out of 64 scenarios (35.9%) with the Robust approach, in 20 scenarios values were equal. The conformity was significantly higher with the Robust approach for both CI_100_ (63 of 64 scenarios, 98.4%) and CI_95_ (64 of 64 scenarios, 100%). The HI was lower for the Robust approach in 43 out of 64 scenarios (67.2%).

As shown in Figure 4, while the advantage of Robust planning in the case of V95%[%] was not statistically significant (*p* = 0.916), the V100%[%] was significantly higher in the Robust planning approach (*p* < 0.001). The conformity was superior for both the 100% isodose (*p* < 0.001) and the 95% isodose (*p* < 0.001) in the Robust approach case (Figure 5). Figure 6 illustrates a superior degree of dose distribution homogeneity within the CTV in cases where the Robust approach was employed (*p* < 0.001).

## 4 DISCUSSION

In the present study, we evaluated the need for the additional facility‐specific margin that may mitigate the effect of uncertainties in beam range and spot position on the robustness of the plan. We hypothesized that the robust planning approach could provide plans with greater robustness to variations in beam positioning parameters compared to the PTV‐based approach. Therefore, the additional offset^13^ equal to 1 mm was introduced in the robustness evaluation to analyze the treatment plans calculated based on the PTV‐based concept and the robust optimization.

The initial plans calculated using the PTV‐based and Robust approaches were quantified in terms of dose coverage, conformity, and homogeneity. These plans were found to be similar in coverage and V100%[cc] conformity (*p* > 0.05) and different in V95%[cc] conformity and homogeneity (*p* < 0.001). The PTV‐based plans were slightly more homogeneous than the Robust plans (HI of 0.011 ± 0.002 vs. 0.028 ± 0.009) but in both cases less than the threshold of 0.05.

**FIGURE 7:**
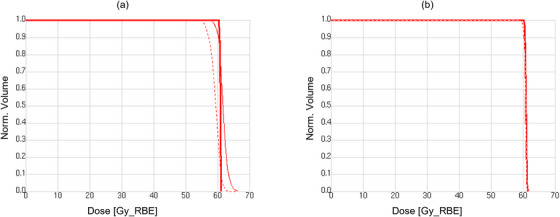
DVH curves illustrating difference in ability of PTV (a) and Robust (b) approaches to preserve CTV coverage. Solid thick line denotes initial plan, solid and dashed thin lines denote setup error scenarios in anterior–posterior directions.

The robustness evaluation was performed with an uncertainty of 3.5%/4 mm, where the total position error was defined as a commonly used treatment margin of 3 mm plus an additional error of 1 mm associated with the facility‐specific beam positioning error (i.e., beam range and spot position uncertainty). The plans calculated using the Robust approach were found to be significantly more robust to a 3.5%/4 mm error than plans calculated using the PTV‐based approach for all metrics used (Figure 7). The Robust planning approach demonstrated a greater tendency to preserve CTV coverage, in accordance with the 100% and 95% prescribed dose, conformity and homogeneity in robustness analysis scenarios.

In this study, the TPS was unable to calculate combined errors.^17^ A total of eight scenarios were considered for each initial plan, comprising six dimensional shifts and two range errors. It is evident that in actual clinical practice, there are numerous potential scenarios that extend beyond the eight scenarios considered in this study. For instance, separate errors can be combined simultaneously, such as a shift in three directions and a range error.^18^, ^19^ The study was conducted using a margin that was specific to the authors’ facility; therefore, the results may not be directly applicable to other PT systems. The present study focuses exclusively on the dosimetric characteristics of PT plans that have been optimized through the application of different approaches, without delving into the associated time costs.

We selected tumors located in a homogeneous region due to the fact that our treatment planning system utilizes analytical dose calculation algorithms. These algorithms calculate dose in tissues with heterogeneities with reduced accuracy compared to the Monte Carlo algorithm. This would introduce additional uncertainties and complicate the assessment of the impact of an additional 1‐mm margin. Therefore, we took tumors in a homogeneous environment to eliminate these uncertainties. Consequently, the exclusion of head and neck tumors, as well as lung tumors, from the study was necessitated by the presence of air cavities and bony structures in these regions.

In the present study, immobile brain tumors far from significant inhomogeneities were selected. The use of robust optimization seems to be able to sufficiently overcome the uncertainties associated with the proton beam position. In the presence of inhomogeneities, it is possible that evaluation of additional margin will be required.

## 5 CONCLUSIONS

The impact of proton beam parameters uncertainty was analyzed in terms of CTV coverage, HI, and CI. The robust optimization approach shows its sustainability to additional 1‐mm beam‐related uncertainty. The sufficiency of the used margin of 3 mm was demonstrated in the context of robust optimization in cases involving tumors that are distant from inhomogeneities and OARs. The contribution of uncertainty associated with beam position should be evaluated individually by each facility. The result of this calculation should be considered when deciding on the PTV margin value. As it has been shown that for some patients the presence of beam position errors can significantly reduce CTV coverage with the prescribed dose, it may be considered appropriate to use an additional margin when planning treatment using the PTV approach. Further research is planned to investigate the effect of beam parameters uncertainties on the dose distribution in a region with strong inhomogeneities, such as the head and neck.

## AUTHOR CONTRIBUTIONS

*Conceptualization*: Vasilii A. Kiselev, and Mikhail A. Belikhin. *Methodology*: Vasilii A. Kiselev, Aleksander P. Chernyaev, and Mikhail A. Belikhin. *Formal analysis*: Rail R. Zamaltdinov, Sergei A. Vladimirkin, Sergei E. Gritsenko, and Aleksey V. Nechesnyuk. *Data acquisition and analysis*: Rail R. Zamaltdinov and, Sergei A. Vladimirkin. *Data curation*: Sergei E. Gritsenko and, Aleksey V. Nechesnyuk. *Writing — original draft preparation*: Vasilii A. Kiselev and, Mikhail A. Belikhin. *Writing — review and editing*: Vasilii A. Kiselev, Mikhail A. Belikhin, Rail R. Zamaltdinov, Sergei A. Vladimirkin, Sergei E. Gritsenko, and Aleksey V. Nechesnyuk. *Visualization*: Vasilii A. Kiselev. *Supervision*: Aleksander P. Chernyaev. All authors critically revised the manuscript, commented on drafts of the manuscript and approved the final manuscript.

## CONFLICT OF INTEREST STATEMENT

The authors declare no conflicts of interest.

## ETHICS STATEMENT

This study complied with the Declaration of Helsinki and was approved by the institutional review board (IRB) (No. 1‐2025). The requirement for written informed consent was waived by the IRB due to the retrospective nature of this study, which involved no more than minimal risk to the subjects. The study used existing medical records and imaging data, and all patient information was anonymized and de‐identified prior to analysis. Furthermore, the waiver did not adversely affect the rights and welfare of the subjects, and the research could not practicably be carried out without the waiver.

## Data Availability

All data generated and analyzed during this study are stored in an institutional repository and can be available upon reasonable request to the corresponding author.
